# The perspective of psychiatric nurses by the European Psychiatric Nurses (Horatio)

**DOI:** 10.1192/j.eurpsy.2024.40

**Published:** 2024-08-27

**Authors:** N. Kilkku

**Affiliations:** European Psychiatric Nurses (Horatio) and European Psychiatric Nurses (Horatio); Faculty of Health Sciences, VID Specialized University, Oslo, Norway

## Abstract

**Abstract:**

In the health care system, nurses are often the biggest professional group and therefore their role is important in the development of service system to meet the current needs of support and help in mental health. Novel solutions are needed, solutions which are not only developed between the professionals, but in collaboration with the people seeking for help, family members, other social networks, and different service providers, like NGOs. Human rights and community-based approaches are guiding this development together with the principles of recovery approach. At the same time there are challenges to overcome, like the shortage of professionals, which also demand new kind of collaboration and solutions to make the field of mental health attractive for future professionals and to support the retention of those who are working in practice at the moment. In the joint symposium the viewpoint of mental health/psychiatric nurses on these issues will be presented.

**Disclosure of Interest:**

None Declared

